# Mapping of Absolute Host Concentration and Exchange Kinetics of Xenon Hyper-CEST MRI Agents

**DOI:** 10.3390/ph14020079

**Published:** 2021-01-21

**Authors:** Martin Kunth, Christopher Witte, Leif Schröder

**Affiliations:** 1 Molecular Imaging, Leibniz-Forschungsinstitut für Molekulare Pharmakologie (FMP), Campus Berlin-Buch, Robert-Roessle-Str. 10, 13125 Berlin, Germany; chris@witte.net.au; 2 Translational Molecular Imaging, Deutsches Krebsforschungszentrum, Im Neuenheimer Feld 280, 69120 Heidelberg, Germany

**Keywords:** MRI, hyperpolarization, CEST, xenon, qHyper-CEST, exchange kinetics

## Abstract

Xenon magnetic resonance imaging (MRI) provides excellent sensitivity through the combination of spin hyperpolarization and chemical exchange saturation transfer (CEST). To this end, molecular hosts such as cryptophane-A or cucurbit[*n*]urils provide unique opportunities to design switchable MRI reporters. The concentration determination of such xenon binding sites in samples of unknown dilution remains, however, challenging. Contrary to ^1^H CEST agents, an internal reference of a certain host (in this case, cryptophane-A) at micromolar concentration is already sufficient to resolve the entire exchange kinetics information, including an unknown host concentration and the xenon spin exchange rate. Fast echo planar imaging (EPI)-based Hyper-CEST MRI in combination with Bloch–McConnell analysis thus allows quantitative insights to compare the performance of different emerging ultra-sensitive MRI reporters.

## 1. Introduction

The search for novel magnetic resonance imaging (MRI) contrast agents is motivated by two interlinked purposes: (a) providing more sensitive agents that can be readily detected at sub mM concentrations, and (b) avoiding toxic side effects such as those known for certain gadolinium (Gd)-based contrast agents (GBCAs) that have led to long-term deposition of Gd ions in tissue and that are subject of an ongoing debate [1,2,3]. To this end, the chemical exchange saturation transfer (CEST) approach with either endogenous substances or synthetic reporters is very attractive because various of these agents are metal free and can be detected with decent sensitivity [4,5,6]. Additionally, MRI sensitivity can be boosted even further by including methods of spin hyperpolarization. Dynamic nuclear polarization (DNP) and para-hydrogen-induced polarization (PHIP) preclude CEST detection because the spin label (most commonly ^13^C or ^15^N) is covalently bound to the molecule/metabolite of interest. It lacks a “bulk pool” that is in chemical exchange with a dilute agent pool that can indirectly provide enhanced sensitivity by accumulating a signal loss from the CEST agent. The hyperpolarizable noble gas ^129^Xe, however, has been used with CEST detection in an approach coined Hyper-CEST because it only engages in transient, non-covalent binding [7]. This combination of two amplification strategies is an emerging technique that provides several benefits. An increasing number of studies also aims to include quantitative MRI aspects as it has been done for ^1^H CEST studies.

Dissolved, chemically inert Xe forms transient complexes with various structures, including proteins, macro- or supramolecular hosts [8,9,10,11], cryptophanes [12,13,14,15,16,17,18,19,20], bacterial spores [21], genetically encoded gas vesicles [22], or perfluoroctyl bromide nanodroplets [23,24,25]. Regarding biogenic binding partners, xenon has been applied as oxygen substitute in assisting structure analysis of oxygen-binding proteins [26,27,28]. Many of these Xe binding sites foster the design of innovative smart Xe biosensors based on highly sensitive Hyper-CEST MRI [29,30,31,32,33,34,35,36,37,38,39]. An important feature of this complex formation is that each of these Xe host systems owns a unique Xe exchange kinetic fingerprint that includes the chemical shift, the exchange rate, the host occupancy, and the binding constant (which is then linked to the gas turnover rate), as well as spin–lattice, and spin–spin relaxation times as one descriptive set of parameters. A given molecular environment impacts each of these parameters. Their complex interplay eventually determines the performance as a Hyper-CEST agent. The net performance for a certain RF saturation power and duration is displayed in so-called *z*-spectra and analyzed through the Bloch–McConnell equations to quantify the kinetics parameters. Even though these spectra plot the detected signal of free Xe versus the frequency offset of the applied RF pulse that saturates magnetization of bound Xe, the analysis goes far beyond analyzing the chemical shift of the CEST pool [40]. For fast screening of multiple xenon hosts in one go, it would be highly desirable to resolve Xe exchange kinetics in different samples through spatial encoding. The spatial variation of these exchange parameters could either be used to characterize hosts in different compartments or environments [18,41], or it can alternatively afford a reliable concentration determination of a Hyper-CEST agent when the response of a reference standard of the same host can be included. As manifested through a nonlinear saturation transfer response, the induced signal loss or CEST effect is not only influenced by the host concentration, but also by the exchange rate and host occupancy. This nonlinearity makes it challenging to determine absolute host concentrations with the CEST method. In fact, ^1^H CEST applications require a rather detailed approach when discriminating the exchange rate from the pool size [42].

Here, we demonstrate a quantitative exchange kinetics mapping method for Xe hosts based on a quantitative Hyper-CEST (qHyper-CEST) concept [43] that disentangles the bound xenon fraction and the exchange rate which are usually represented in the CEST effect intensity as a product term. Using these maps allows to achieve absolute host concentration mapping [44]. Our proposed framework also greatly facilitates the comparison of host candidates for Hyper-CEST as an emerging class of contrast agents that is still seeking for highly efficient hosts en route to in vivo translation. It will complement similar efforts that have been implemented for ^1^H CEST screening [45].

When comparing the exchange kinetics of different type of Xe host systems (e.g., CrA-ma, cryptophane-A monoacid, in DMSO, CrA-ma in H${}_{2}$O, and cucurbit[6]uril in H${}_{2}$O), there are large differences in the quantitative exchange kinetics between these different systems [46]. Remarkably, studying the identical Xe host CrA-ma in two different solvents—DMSO and H${}_{2}$O—showed entirely different exchange kinetics for a given temperature [47]. This behavior strongly suggests the idea of a characteristic Xe host-specific exchange kinetics NMR fingerprint that is assigned to each system. Certain exchange kinetics aspects have been investigated. For instance, large differences in the Xe release rate from different hosts and in different solvents are also known through changes in the observable effective transverse relaxation time, $T_{2,\mathrm{eff}}$, and can be compared in Swift–Connick plots [46]. This approach here extends the Xe host classification capability beyond the chemical shift dimension and binding constant as derived from one-dimensional NMR spectra in the early literature [40]. One could summarize such a fingerprint using an *n*-tuple where the entries consist of all exchange kinetic parameters of the particular Xe-host system. A prototype for a 2-spin pool system fingerprint could comprise the following parameters:

$$\langle \;T_{1}^{A}\;|\;T_{2}^{A}\;|\;\Delta \omega \;|\;k_{\mathrm{BA}}\;|\;f_{B}\;\left|\;\beta \;\right|\;K_{A}\;\rangle (T,[\mathrm{Xe}])$$

This is a function of temperature *T* and of the applied Xe concentration [Xe]. We demonstrate in this study that a careful analysis of saturation transfer spectra acquired with a set of different RF saturation pulse settings allows determination of all these parameters by using the full Hyper-CEST (FHC) solution [48] with reasonable computational effort even for pixelwise analysis that has not been accomplished before in Xe MRI.

## 2. Results

### 2.1. Mapping of Xenon Exchange Kinetics

**Figure 1:**
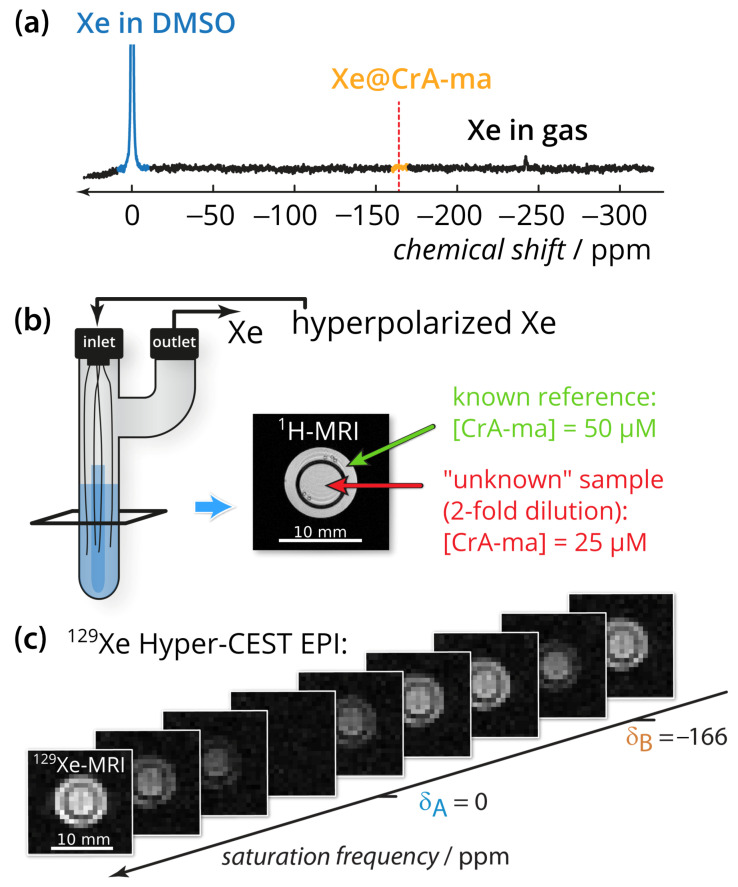
Experimental setup. (**a**) Direct ^129^Xe NMR spectrum with 100-fold zoom of 16 scans of the sample described in subfigure (**b**). The red dashed line indicates the chemical shift of the Xe@CrA-ma in DMSO resonance. (**b**) Two-compartment phantom including glass capillaries and sample (blue). A gas mixture of freshly hyperpolarized Xe was bubbled into the sample via the inlet and is vented via the outlet. The outer compartment (green arrow in the axial ^1^H-MRI) contained the known reference concentration with [CrA-ma] = 50 μM, whereas the inner compartment (red arrow) carried the “unknown” concentration (in fact, the 2-fold dilution; 25 μM). (**c**) ^129^Xe Hyper-CEST-EPI axial images series with respect to the saturation frequency. Whereas complete saturation occurs at the saturation frequency of free Xe at δ_A_, only a fraction is saturated at the CrA-ma-bound Xe δ_B_. The pixel-wise qHyper-CEST analysis was done for saturation with *B*_1_,*t*_sat_ = {3.3,5; 5.6,10; 2.2,15} μT, s.

The quantification method was applied to CrA-ma in DMSO at two different concentrations (Figure 1b). DMSO was chosen as the solvent because it allows demonstrating the method for a system with an already weak binding constant (previously published as 38 M^−1^) where only ≈10% of the binding sites are actually occupied [43,47]. Using DMSO illustrates the usefulness even for promising Xe hosts that have a low binding constant but favorable exchange rates. Nevertheless, we expect the method to also provide important insights for comparative studies in aqueous conditions. Conventional direct Xe NMR spectroscopy was not able to identify CrA-ma in DMSO at moderate signal averaging (Figure 1a; average of 16 scans) and thus illustrates the need for more sensitive techniques, particularly when spatial encoding is included to compare multiple samples side-by-side. By taking advantage of imaging using echo planar imaging (EPI) for hyperpolarized Xe [17], we could indeed analyze both samples side-by-side for an array of different saturation frequencies (see image series in Figure 1c; a movie of this images series is provided in Appendix A).

**Figure 2:**
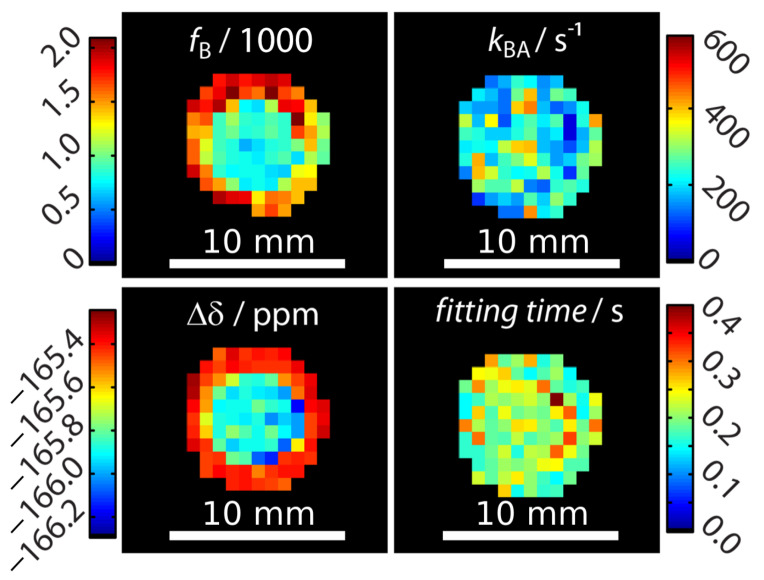
Quantitative results for mapping xenon exchange kinetics of CrA-ma in DMSO at *T* = 295 K. Pixel-wise fitting results of the qHyper-CEST concept using the full Hyper-CEST (FHC) solution [48]. The following parameters were mapped: the ratio of bound and free Xe, $f_{B}$, the Xe exchange rates, $k_{\mathrm{BA}}$, the relative chemical shifts, Δδ, and total fitting times per pixel.

Signal fitting was done using the FHC solution [43,48]. Instead of deriving the signal from an entire region-of-interest (ROI) as done earlier [43,47,49], a pixel-wise fit was performed after applying a mask that excludes pixels outside the phantom with no signal. The *z*-spectra quality of two pixels (one in the outer compartment and on in the inner compartment) can be seen in Appendix B. Each pixel carries quantitative information regarding the ratio of bound and free Xe, *f*_B_, the Xe exchange rate, *k*_BA_, the relative chemical shift, Δδ, and the total fitting times per pixel. These are displayed as corresponding parameter maps (Figure 2). Mapping of $f_{B}$ and Δδ clearly reproduced the geometric shape of the two-compartment phantom. In contrast, the exchange rate showed a homogeneous distribution across the double phantom, thus illustrating its independence of the host concentration. All parameters were in excellent agreement with previously reported values derived from ROI-averaged data: chemical shift difference Δδ = (−166.69 ± 0.02) ppm, the exchange rate *k*_BA_ = (290 ± 20) s^−1^, and the host occupancy β = 9% [43,47,49]. The map of the fitting time shows that the FHC solution efficiently converged on average in less than 300 ms per pixel to the global minimum (Figure 2). This is very fast in comparison to evaluating the Bloch–McConnell equations with either the matrix inversion method [50] or exponential matrix method [51] as the FHC solution had been demonstrated to be about 30–70-fold faster for data with lower noise level [43]. It nicely demonstrates the advantages of FHC with regard to larger data sets that maintain all the exchange kinetics parameters in a spatially resolved manner. Fitting the data with using the full Bloch–McConnell equations for *N* relevant pixels (in this case: N≈100) would have taken at least about *N* times 30 s (i.e., ~3000 s = 50 min; the time of 30 s per fit was required in a previous study with smoother spectra due to ROI-averaging [43]); however, the analysis took about 30 s using the FHC solution. Only the FHC approach provides realistic postprocessing times for pixel-wise fitting of image series with even much larger matrix sizes.

An unexpected result was that the chemical shift separation of both Xe pools differed by 0.6 ppm between the inner and the outer compartment. This was significantly larger than the error range of this measurement. We first hypothesized that this effect could potentially arise from the different CrA-ma concentrations used in this experiment [43]. As a control, a two-compartment sample with identical CrA-ma concentrations in the inner and outer compartment was investigated but still showed a nearly identical chemical shift difference of 0.7 ppm between both compartments (see Appendix C). We therefore assigned this discrepancy to the rather pronounced sensitivity of the Xe@CrA-ma resonance to a temperature difference between both compartments [52,53] as an imperfection from the (indirect) heating setup of the variable temperature unit that we used for temperature control. This is also consistent with the observation that the saturation response around 0 ppm is practically identical for both compartments (and thus susceptibility effects near the glass walls can be excluded as explanation), whereas the response from the CEST pool appears shifted (see data in Appendix B). This signal from bound Xe is known to shift stronger with temperature than that of free Xe [52,53]. We observed that this changed the chemical shift to a measurable extent, but the exchange rate varied only insignificantly such that it was not observable in Figure 2.

### 2.2. Absolute Xenon Host Concentration Mapping

**Figure 3:**
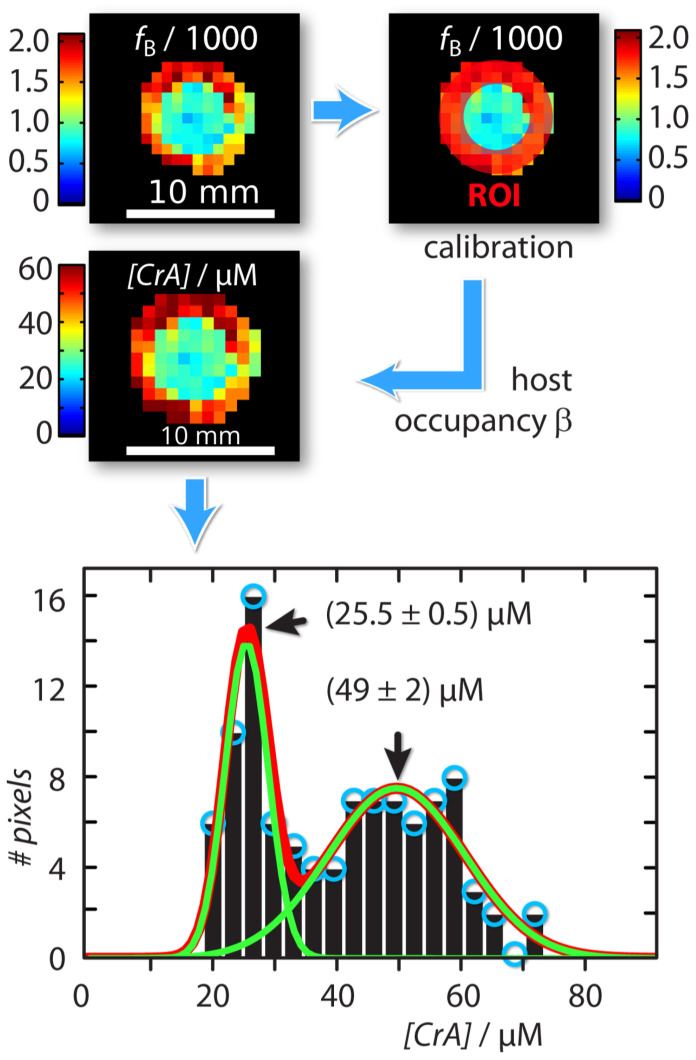
Workflow of absolute concentration mapping starting with the ratio of bound and free Xe, $f_{B}$, at the top left: The host occupancy, β, was calibrated with the Hyper-CEST response produced by the known reference sample in the outer compartment (red region-of-interest (ROI)) according to $\beta_{\mathrm{cal}}$ = $f_{B}\;\times$ ([Xe]/[CrA-ma]_OC,known_) = 9%. The unknown CrA-ma concentration in the inner compartment was then calculated with [CrA-ma]_IC,unknown_ = ($f_{B}$/$\beta_{\mathrm{cal}}$) × [Xe]. The Gaussian line fitted histogram of the [CrA-ma] map identified two different concentration populations around (25.5 ± 0.5) μM and (49 ± 2) μM (green solid lines; linear combination: red solid line).

An absolute xenon host concentration determination was possible by including quantitative information from one of the compartments as an internal standard with known host concentration. Assuming identical exchange kinetics, this can be used to characterize a sample with unknown host concentration. For this, we exploited the property that both the host occupancy β, and the affinity constant $K_{A}$ (which are related by β = [Xe] $\times \;K_{A}$/(1 + [Xe]$\;\times \;K_{A}$) [43] are constant in DMSO over a concentration range of CrA-ma starting from 1 μM up to 150 μM [43]. This is because Xe is always redelivered to maintain chemical saturation. As a result, we can calibrate the Xe host occupancy from the known sample as β_cal_ = *f*B× ([Xe]/[CrA-ma]_known_). This calculation is based on the relationship *f*_B_([host_tot_]) = β× ([host_tot_]/[Xe]) as given in [43]. We can then calculate the unknown host concentration in the inner compartment by [CrA-ma]_IC,unknown_ = (*f*_B_/β_cal_) × [Xe] (see Figure 3). This yields a map with concentration information for each pixel. The histogram analysis using Gaussian distribution for this concentration map showed two populations around (25.5 ± 0.5) μM and (49 ± 2) μM (Figure 3). This was in excellent agreement as the test sample in the inner compartment was in fact a 2-fold dilution of the outer reference compartment.

### 2.3. Limitations

When multiple samples with different host concentrations are under investigation, the saturation pulse strengths should be chosen accordingly. This ensures that the CEST effect responses remain within a reasonable dynamic range of the Xe depolarization rate [43]. Further, when the CEST response for one type of host is more narrow than that of the simultaneously measured one in the neighboring compartment, then the saturation frequency step size should be adjusted accordingly. The method is thus limited by the performance and dynamic range of qHyper-CEST, i.e., the minimum amount of host material has to be such that three different RF saturation pulse strengths $B_{1}$ produce three uniquely different Xe depolarization rates. A minimum efficient *f*_B_ in this case thus requires at least [CrA-ma]_min_ ≈ 1 μM. Regarding high concentrations, the CEST response can be reduced accordingly for high host concentrations by reducing the RF saturation pulse parameters to still match a certain dynamic range and to avoid complete saturation. However, there is no motivation to use Hyper-CEST when excessive (>200 μM) CrA-ma is dissolved because this generates a directly detectable Xe@CrA-ma peak in ^129^Xe NMR spectroscopy. The important prerequisite for the absolute quantification method proposed in this present work is that both the Xe host occupancy β (in %) and thus also the affinity constant $K_{A}$ are constant, i.e., the chemical conditions for the affinity must be the same in both samples. These parameters clearly depend on the host, the solvent, and the temperature that were chosen for a particular study and must be consistent when comparing absolute numbers.

This condition can be easily fulfilled in in vitro experiments where the repetitive bubbling with the Xe gas mix ensures that the solution is always chemically saturated with the gas according to the Ostwald coefficient. The fraction of bound Xe is determined by the binding constant and remains unchanged during the experiment. A stable baseline in the *z*-spectra is the experimental confirmation that a reproducible steady-state magnetization has been reached after each gas delivery for the individual acquisitions. Another aspect to consider is a variable protonation at the host portals that might impact the Xe affinity in different pH. While the concept of pH is irrelevant in the DMSO conditions used here, such pH-depending protonation might be a relevant aspect in aqueous solutions and should be adjusted with appropriate buffer systems for comparison studies.

Overall, this exchange parameter mapping technique is (for now) only reliable and attractive for in vitro applications where different hosts for reversible binding of Xe shall be compared side by side with two clearly defined spin pools. Chemical engineering that aims to tune the exchange rate is a prime example of this. Translation to biological systems requires more detailed separation of the individual components as a live cell assay would clearly increase the number of involved spin pools (Xe and host can both be present in different micro-environments). In vivo applications would also be limited until there is further knowledge available regarding the complete exchange network of all participating pools, the occupancy of hosts, and the solubility of Xe and how this can be compared in different tissues and if any correction methods can be applied to obtain absolute quantitative numbers.

It is noteworthy that a quantification based on this approach requires a spectrally resolved CEST-response because the math framework otherwise fails to derive the exchange kinetics. Therefore, hosts providing exchange kinetics that appear fast on the NMR timescale and only cause a broad MT effect (e.g., pillar[5]arenes [38]) cannot be characterized with this method. Preliminary tests on pillar[6]arene-based hexagonal boxes also showed no CEST response.

## 3. Discussion

CEST agents come with the advantage that they provide an adjustable contrast by choosing well-defined RF saturation conditions. Their concept thus differs from the conventional “passive” relaxation agents whose impact on the detected bulk spin pool is not actively driven by the applied RF irradiation. This direct manipulation through the observer also allows to interrogate the system of exchange-coupled spins by well-defined, complementary saturation conditions and to analyze the exchange kinetics based on the different spectral responses to these RF conditions. The use of ^129^Xe in CEST studies has certain advantages over exchanging ^1^H nuclei: First, it allows combination with spin hyperpolarization [7], and thus includes another type of sensitivity enhancement beside the signal transfer from a dilute onto an abundant pool through saturation transfer. Even more important in terms of analytical aspects is the fact that the detailed quantitative analysis of the spectral CEST response is significantly simplified for exchanging Xe compared to other “flavors” of CEST with protons. Xe has unique NMR properties that include (a) a rather inefficient relaxation due to its inert character and (b) a large chemical shift range caused by its large electron cloud. Therefore, it is sensitive to its chemical environment predominantly through its Larmor frequency but not through its relaxation behavior. This is an ideal combination for CEST measurements because the intrinsic relaxation that is counteracting the actively driven CEST response can be neglected and the time evolution of the magnetization $M_{z}\left(t\right)$ in the exchange-connected pools can be easily separated when solving the Bloch–McConnell equation (i.e., for most saturation cases, the RF irradiation can be assumed as rather selective and it affects only one pool). These aspects have led to the simplified quantitative Hyper-CEST analysis (qHyper-CEST) [43,48].

**Figure A3:**
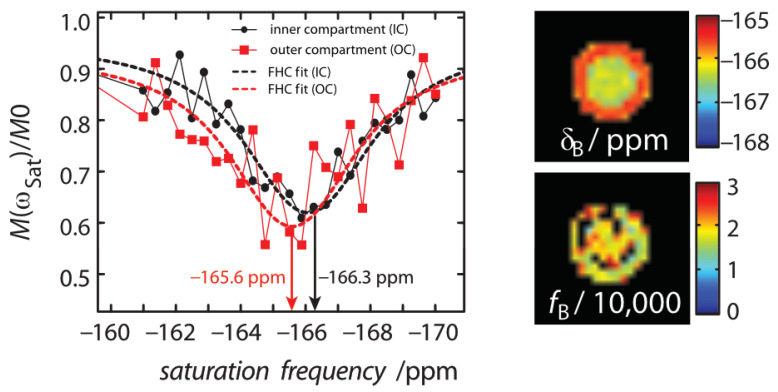
For an experimental setup of equal concentrations of CrA-ma in the inner and outer compartment (IC and OC) of the double bubbling phantom ([CrA-ma${}_{\mathrm{IC}}$] = [CrA-ma${}_{\mathrm{OC}}$] = 10 μM), we observed a chemical shift difference of the CEST resonance, $\delta_{B}$ map, while the Xe in DMSO resonance, $\delta_{A}$, remained constant. The equal concentrations of CrA-ma in the IC and OC was confirmed by the map of the ratio of bound and free Xe, $f_{B}$, that showed no significant difference (bottom right). The black squares within the phantom correspond to values close to zero. They originate from partial volume effects because of FHC fitting of the glass wall of inner 5 mm NMR tube. The fit result of the $\delta_{B}$ map was not compensated by another fit parameter. Taking into account the very sensitive shift of Xe@CrA-ma with temperature, we assigned this difference in chemical shift to a temperature difference between both compartments.

Such reduced computational effort is the prerequisite for performing a pixelwise analysis in setups that contain multiple samples. Modern polarizers for the production of hyperpolarized ^129^Xe through spin exchange optical pumping provide enough starting magnetization that spatial encoding schemes can be applied [54,55], even for Xe that is dissolved at μM concentrations in solvents or tissue. In this study, a sub-mm in-plane resolution still yields CEST spectra that are easy to fit with the analytical tool (see Figure A3). However, a highly reproducible Xe delivery should be ensured to reduce the shot-to-shot noise while the spectral dimension along the CEST spectrum is encoded step by step [56]. In unlocalized Hyper-CEST measurements, our system achieves signal fluctuations of less than 1% [56].

The entire set of fitting parameters as summarized in the above-mentioned tuple provides information on both the solvent NMR properties and the exchange kinetics. Importantly, the fraction of bound Xe can be rather small (≤0.2% in this case) and is still providing quantitative insights. The Xe spins spent most of the time in the bulk pool where they simply undergo slow $T_{1}$ relaxation towards equilibrium with a vanishing longitudinal magnetization (the conditions used here provide a $T_{1}$ of Xe in DMSO of ∼120 s [43]). This is represented by a decaying baseline in the spectra for increasing saturation time. The decay constant of the transverse magnetization $T_{2}^{A}$ is always present and, together with the $B_{1}$ amplitude, determines the width of the direct Xe@solution response at 0 ppm.

The Δω and the remaining fit parameters describe the entire exchange kinetics picture and the classification of fast ($\Delta \omega \ll k_{\mathrm{ex}}=k_{\mathrm{BA}}+k_{\mathrm{AB}}\approx k_{\mathrm{BA}}$) vs. slow exchange ($\Delta \omega \gg k_{\mathrm{ex}}$) on the NMR time scale. The large chemical shift range of Xe often provides conditions for slow exchange. Some host structures, however, provide intermediate exchange, but the qHyper-CEST analysis has proven to also work for such systems [57].

The advantage of qHyper-CEST analysis is that the exchange rate $k_{\mathrm{BA}}$ and the pool size $f_{B}$ can be disentangled. This is challenging for other CEST systems where the benefits from the above-mentioned simplifications regarding the solution of the Bloch–McConnell equations do not apply. Without these benefits, a larger variation range of saturation time or power is necessary to obtain absolute exchange rates, even if the agent concentration is known [42,58]. The amount of bound Xe is easily accessible in our approach and its combination with the overall host concentration known from sample preparation yields the fractional host occupancy, β. This parameter is used to define the gas turnover $\beta \times k_{\mathrm{BA}}$, a quantity that is useful for comparison of host systems [47] and their performance in different solvents. As described above, β is also critical for applying the calibration with the internal standard.

The concentration mapping identified the host concentration relative to the internal standard with an error of ~2%. Its histogram representation illustrates that the internal concentration standard actually exhibits a wider scattering. We ascribe this observation to the fact that the reference volume may contain a large number of pixels that are affected by partial volume effects and include some void signal contributions from the glass wall. This causes a relative large scattering compared to the “test volume” in the center compartment that has a more clean definition regarding which pixels should be included. As long as the saturation response remains within the dynamic range and does not approach complete saturation, the (nonlinear) relationship and between the CEST amplitude and the host concentration follows the predicted theoretical behavior introduced by Zaiss et al. [48]. This allows to directly link a change in the CEST response to a concentration change as experimentally demonstrated by Döpfert et al. [37] where a linear decrease in the host pool of 16 to 0 μM was clearly confirmed during the onset of an enzymatic conversion. We thus focused in this study on one exemplary scaling factor (twofold dilution) while it has been shown that the Hyper-CEST technique can follow a wider range.

Overall, we envision that the comprehensive Xe-host exchange kinetics NMR fingerprint can contribute to sense complex in vitro scenarios. It could serve as input for an *N*-pool expansion of the Bloch–McConnell equations to analyze the behavior of CEST agents in a complex environment. The technique shall be helpful for various in vitro characterization of Xe host systems that are currently under investigation for the design of Xe biosensors en route to preclinical applications [59,60].

## 4. Materials and Methods

### 4.1. Sample Preparation

Samples were prepared by dissolving cryptophane-A mono-acid (CrA-ma, provided by Kang Zhao, Tianjin University, China) into dimethyl sulfoxide (DMSO) at room temperature at a concentration of [CrA-ma] = 50 μM. Whereas the outer compartment of two nested NMR tubes (a two-compartment phantom; see Figure 1b) was filled with this solution of 50 μM of CrA-ma in DMSO, the inner compartment contained an “unknown” 2-fold dilution.

### 4.2. Hyperpolarization and ^129^Xe Delivery

Circa 25 % Xe spin hyperpolarization of a 2% Xe gas mix {2, 10, 88}-vol.% of {Xe, N${}_{2}$, He} with ^129^Xe (natural abundance: 26.4%) was obtained with a continuous-flow (0.35 SLM) custom-designed polarizer [61] via spin exchange optical pumping with rubidium atoms. The electrons of rubidium were excited by a 150 W cw-laser (795 nm, 0.5 nm bandwidth, QPC Lasers) at a total pressure of *p* = 4.5 atm. Before signal acquisition, the samples were bubbled for 13 s at a flow rate of 0.1 SLM, followed by a 2 s delay in order to allow the bubbles to collapse. Assuming Xe saturation, the Xe concentration in DMSO in chemical equilibrium (which is reached within the first 2–3 repetitions of Xe gas delivery), was [Xe] = 2340 μM ([Xe] = L×p× Xe${}_{\mathrm{pc}}$/(0.0254 L/mM), with the Xe Ostwald solubility coefficient in DMSO L = 0.66 L/atm and Xe${}_{\mathrm{pc}}$ = 0.02).

### 4.3. NMR Experiments

NMR experiments were done at $|{\vec{B}}_{0}|$ = 9.4 T with an NMR spectrometer (Bruker Biospin, Ettlingen, Germany) equipped with gradient coils for imaging and a variable temperature unit. All samples were measured at room temperature (*T* = 295 K). For excitation and detection, a 10 mm inner diameter double-resonant probe (^129^Xe and ^1^H) was used. The $B_{1}$ field inhomogeneities can significantly affect the CEST quantification and these must be known. However, as shown in [43], such inhomogeneities were negligible for our micro-imaging system. Moreover, the $B_{0}$ field homogeneity is sufficient enough throughout the sample to achieve a line width of dissolved, unbound Xe of ca. 1 Hz. Further field map correction is thus not required. ^129^Xe Hyper-CEST images were obtained with a ^129^Xe Hyper-CEST echo-planar imaging [17] pulse sequence with the following parameter settings: Fourier acceleration: 1.68 (a feature of Cartesian *k*-space sampling where only 32/1.68 = 19 lines of the full *k*-space have been acquired [17]); double sampling (an EPI-specific feature [17]); echo time: 5.7 ms; acquisition time: 19.8 ms; (no smoothing filter was applied to the images); field of view: 20 × 20 mm${}^{2}$; matrix size: 32 × 32; in plane resolution: 625 μm, slice thickness: 20 mm. The saturation pulse strengths and durations used are given in the figure captions.

### 4.4. Data Fitting

qHyper-CEST analysis was performed based on the imaging series that yielded pixel-wise *z*-spectra from different saturation parameters (see figure captions). A mask was applied to exclude pixels outside the phantom area. All calculations and fitting routines were implemented and performed in Matlab 7 (The Mathworks, Natick, MA, USA) on a standard desktop PC (64 bit, 8 cores each at 2.80 GHz, 8 GB RAM) as described in [43]. Pixel-wise fitting time was recorded using tic and toc in Matlab.

## 5. Conclusions

By extending the qHyper-CEST concept to imaging with pixel-wise analysis, we mapped the unique fingerprint of a specific Xe-host system within a particular chemical environment. We could demonstrate the determination of absolute host concentrations as an extended analysis of the previously introduced FHC approach. Our results provide faster insights into the Xe-host binding kinetics since multiple Xe-host systems can be screened at once.

This quantitative MRI approach demonstrates the potential of the FHC approach for data sets with increased spatial resolution compared to initial qHyper-CEST analysis. It will be of particular interest for in vitro tests of newly developed Xe biosensors but also for quantitative physical chemistry studies of Xe-binding host structures where the simultaneous detection of multiple samples overcomes the challenges of introducing hyperpolarized spin reporters.

## Figures and Tables

**Figure A1:**
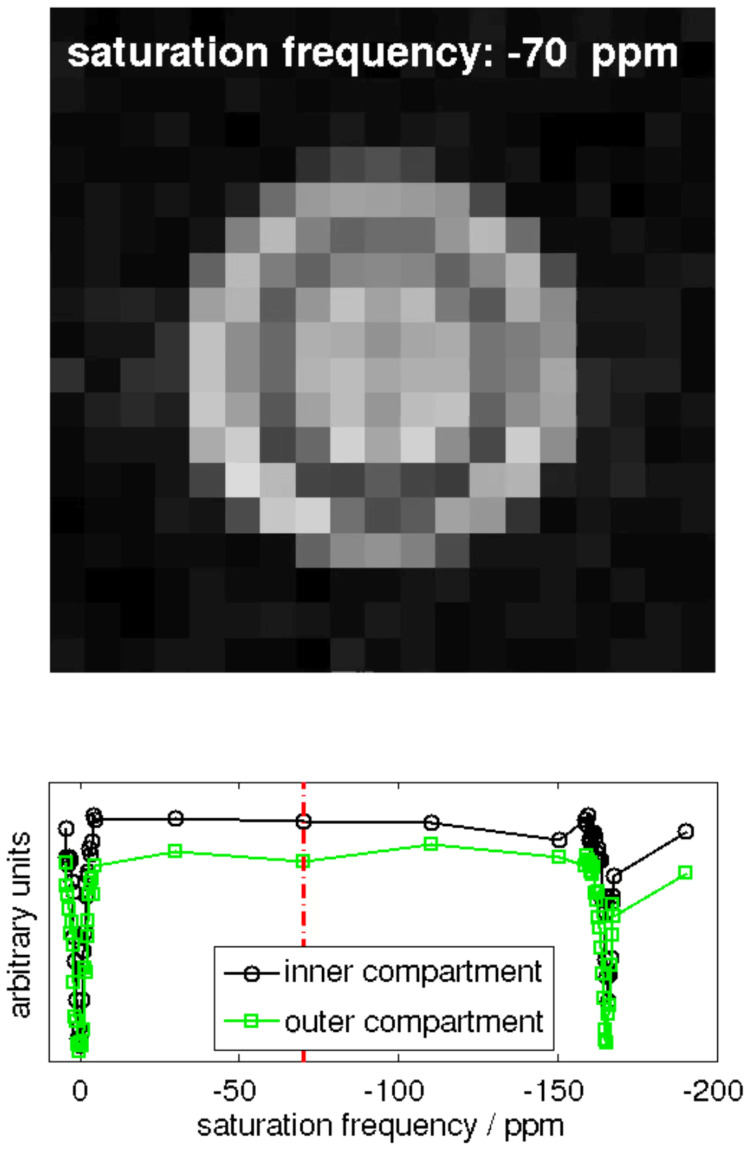
Snapshot of the animation of the ^129^Xe MR image series obtained with a Hyper-CEST EPI pulse sequence. The video itself can be found in the online Supplementary Materials.

**Figure A2:**
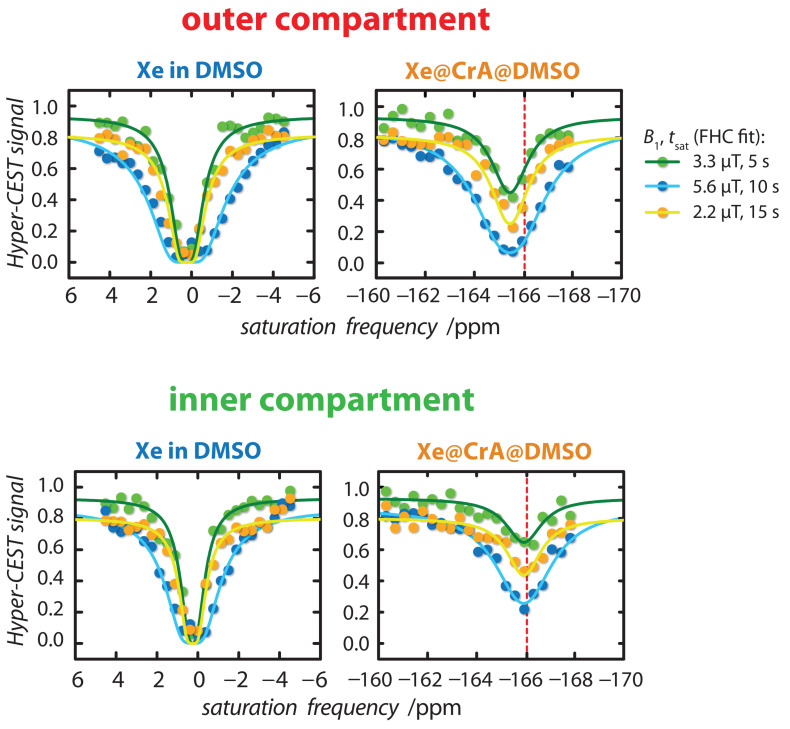
Hyper-CEST *z*-spectra for data processing exemplary for one pixel in the outer (**top**) and inner compartment (**bottom**) of the double bubbling phantom. The Hyper-CEST *z*-spectra (dots) were globally fitted (a set of three spectra for each pixel) with the full Hyper-CEST (FHC) model (solid lines) for $B_{1}/t_{\mathrm{sat}}$ = {3.3/5 (green), 5.6/10 (blue), 2.2/15 (orange)} μT/s. The red dashed line at the Xe@CrA-ma resonance marks a fix line at −166 ppm to illustrate the chemical shift between the inner and outer compartment. This shift is above the uncertainty of the fit result, i.e., for the inner compartment $\Delta \delta_{\mathrm{IC}}=\pm 0.09$ ppm and the outer compartment $\Delta \delta_{\mathrm{OC}}=\pm 0.04$ ppm.

## Data Availability

The data presented in this study are available within the article or on request from the corresponding author.

## Supplementary Materials

The following are available at https://www.mdpi.com/1424-8247/14/2/79/s1, Video S1: Animation of Xe MR image series obtained with a Hyper-CEST EPI pulse sequence.

## Abbreviations

The following abbreviations are used in this manuscript:

CEST	chemical exchange saturation transfer
CrA-ma	cryptophane-A monoacid
DMSO	dimethyl sulfoxide
DNP	dynamic nuclear polarization
EPI	echo planar imaging
FHC	full Hyper-CEST
FOV	field of view
GBCA	Gadolinium-based contrast agent
Gd	Gadolinium
Hyper-CEST	linear dichroism
MRI	magnetic resonance imaging
NMR	nuclear magnetic resonance
qHyper-CEST	quantitative chemical exchange saturation transfer with hyperpolarized xenon
ROI	region of interest
Xe	Xenon

## Appendix A. EPI Image Series

Hyper-CEST EPI [17] and corresponding *z*-spectra (exemplary for one pixel in the inner and one pixel in the outer compartment of the double bubbling phantom is shown in Figure A1.

## Appendix B. Details of Pixel Wise z Spectra

The pixel-related *z*-spectra of two pixels (on in the inner compartment, and one in the outer compartment) disclosed the shift in chemical shift difference (Figure A2).
